# Doing Time: Experiences of Care Home Residents in Germany During the Early Phase of the COVID-19 Pandemic

**DOI:** 10.1093/geroni/igab046.2037

**Published:** 2021-12-17

**Authors:** Frank Oswald, Arthur Schall, Johannes Pantel, Miranda Leontowitsch

**Affiliations:** 1 Goethe University Frankfurt, Frankfurt am Main, Hessen, Germany; 2 Goethe University Frankfurt, Frankfurt, Hessen, Germany; 3 Goethe University Frankfurt, Frankfurt / Main, Hessen, Germany

## Abstract

Residents of care homes across the globe are affected by the spread of SARS-CoV-2 as they have been identified as a high-risk group and because they experienced strict social isolation regulations during the first wave of the pandemic. Social isolation of frail older people is strongly associated with negative health outcomes. The aim of this research project was to investigate how residents in care homes experienced social isolation during the first phases of contact ban in Germany. This paper draws on structured interview data collected from 22 residents in two care homes during early June 2020 in Frankfurt/Main. The findings show that their experiences were shaped by three factors: care home staffs’ approach to handling the contact ban; biographical sense of resilience; and a hierarchy of life issues. The findings highlight the importance of locally specific response mechanisms in care homes, and the need to contextualize residents’ experiences.

